# Serology, infection, and clinical trachoma as tools in prevalence surveys for re-emergence of trachoma in a formerly hyperendemic district

**DOI:** 10.1371/journal.pntd.0009343

**Published:** 2021-04-16

**Authors:** Michelle Odonkor, Fahd Naufal, Beatriz Munoz, Harran Mkocha, Mabula Kasubi, Meraf Wolle, Sheila West

**Affiliations:** 1 Dana Center for Preventive Ophthalmology, Wilmer Eye Institute, Johns Hopkins University, Baltimore, Maryland, United States of America; 2 Kongwa Trachoma Project, Kongwa, Tanzania; 3 Department of Microbiology, Muhimbili University of Health and Allied Sciences, Dar es Salaam, Tanzania; RTI International, UNITED REPUBLIC OF TANZANIA

## Abstract

**Background:**

To eliminate trachoma as a public health problem, countries must achieve a district-level prevalence of trachomatous inflammation—follicular (TF) <5% in children ages 1–9 years. Re-emergence of TF could trigger additional rounds of mass drug/antibiotic administration (MDA), so accurate tools for use in surveys assessing trachoma prevalence are essential.

**Methodology & principal findings:**

We surveyed 2401 children ages 1–9 years from 50 villages in Kongwa, Tanzania, 2 years post-MDA and 1.5 years after an impact survey found TF <5% in the same villages. Our survey included multiple tools: clinical determination of TF, Cepheid testing for *Chlamydia trachomatis* infection, and testing for anti-pgp3 antibodies via multiplex bead array. Photographs of the upper tarsal conjunctiva were taken in a subset of children to corroborate the field grades.

Overall TF prevalence in 1–9 year olds was 7.1% (*95% CI*: 5.6%-8.9%), which decreased with age (p = <0.0001). TF prevalence by village was heterogeneous, with 19 villages having TF <5% and 16 villages having TF >10%. There was a strong correlation between field and photo grading of TF (kappa = 0.69; *95% CI*: 0.60–0.78) and between TF and infection, with 21.5% of TF-positive children also testing positive for infection, as compared to only 1.6% of TF-negative children (p = 0.0010). Overall seroprevalence was 18.2% (*95% CI*: 14.8%-22.1%), which increased with age (p = <0.0001). Notably, 1–2 year olds, who were born after the cessation of MDA and theoretically should not have had exposure to *C*. *trachomatis* in the absence of transmission, had an average seroprevalence of 6.7%.

**Conclusions & significance:**

Field TF prevalence, supported by photographic review and infection data, suggested re-emergence of trachoma in Kongwa. Moreover, seropositivity in the children born after cessation of MDA indicated exposure to *C*. *trachomatis* despite a previous survey finding of TF <5%. Examining seropositivity in specific age groups expected to have limited exposure to *C*. *trachomatis* can be used to detect re-emergence.

## Author summary

Trachoma, a chronic conjunctivitis caused by the bacterium *Chlamydia trachomatis*, is the leading infectious cause of blindness worldwide. To eliminate trachoma as a public health problem, countries must achieve trachomatous inflammation—follicular (TF) prevalence <5% in children ages 1–9 years in each endemic district, as measured by two surveys conducted after cessation of mass drug/antibiotic administration (MDA). An impact survey in Kongwa District 6 months post-MDA found TF <5%. We conducted another survey 2 years post-MDA in the same villages as the impact survey using multiple tools: assessment of TF (including via photographs of the upper tarsal conjunctiva), infection with *C*. *trachomatis*, and seropositivity to chlamydial antigen pgp3. We found a TF prevalence of 7.1%, and 21.5% of TF-positive children tested positive for infection (p = <0.0001). Review of photographic images showed good agreement with field grading. Seropositivity was 18.2% and increased with age (p = <0.0001). The youngest children—who were born after the last round of MDA and in theory should not have had much exposure to infection—had a seropositivity of 6.7%, indicating community transmission during the 2 years post-MDA. All tools pointed to re-emergence of trachoma and supported the feasibility of using targeted serologic markers as a robust surveillance tool.

## Introduction

Trachoma is a chronic conjunctivitis caused by repeated episodes of infection with *Chlamydia trachomatis*. As the leading infectious cause of blindness in the world, trachoma has caused visual impairment or blindness in about 1.9 million people and is responsible for 1.4% of all cases of blindness worldwide [1]. A country must achieve the following criteria to demonstrate elimination of trachoma as a public health problem: (i) prevalence of trachomatous trichiasis unknown to the health system of <0.2% in adults ages 15 years and older at the district level, as well as having a strategy for identifying and managing incident cases of trachomatous trichiasis, and (ii) prevalence of trachomatous inflammation—follicular (TF) of <5% in children aged 1–9 years old at the district level [2,3]. Elimination is confirmed by two district-level population-based surveys, conducted at least two years apart in the absence of mass drug/antibiotic administration (MDA), that have met both metrics [3].

There is ongoing debate over the best way to conduct surveillance for potentially re-emergent TF. Clinical determination of TF has been criticized because of grading imprecision and concern of overcalling TF especially when disease prevalence is low. For example, there have been reports of TF prevalence >5% in communities with zero or near-zero prevalence of infection [4,5]. A test of infection alone is equally problematic as there are no guidelines on what prevalence of infection constitutes re-emergence. There is evidence from research and district surveys where the presence of a low level of infection, or infection in a setting that did not favor ongoing transmission, was insufficient to cause long-term re-emergence of trachoma [6,7]. Serologic testing for chlamydial antibodies is another potential tool and may provide a better understanding of ongoing disease transmission, depending on the district’s history and the age groups of interest. Serology may reflect cumulative exposure over time, although seroreversion is an issue and is more common in villages where trachoma is low [8]. Data are needed on all of these tools from districts that are potentially at risk of re-emergent trachoma to understand patterns that might detect re-emergence of TF.

A 2017 impact survey conducted by the Tanzanian national Neglected Tropical Diseases Control Program (NTDCP) 6 months post-MDA found a TF prevalence of <5% in a random sample of children aged 1–9 years old from 50 villages randomly selected in Kongwa District, Tanzania. We hypothesized that the prevalence of trachoma in the district 2 years after MDA would be less than 5% in children ages 1–9 years. We report data from the same villages 2 years post-MDA, from August to December 2018, where we used multiple tools to examine the risk of re-emergence. These survey tools included prevalence of TF as determined by field grading and by grading of photographs taken of the children’s upper tarsal conjunctivae, testing for infection, and testing for antibody positivity to *C*. *trachomatis* antigen pgp3. The goals of our study were twofold: 1) to determine the prevalence of TF and to show the correlation between field grading of TF and grading of photographs of TF, as well as the correlation between TF and infection, and 2) to determine the overall and age-specific rates of seropositivity in Kongwa District, Tanzania.

## Methods

### Ethics statement

The study protocols and procedures were reviewed and approved by the Johns Hopkins School of Medicine Institutional Review Board and the National Institute for Medical Research in Tanzania. We obtained written informed consent from the guardians of study participants, as well as additional verbal assent from participants aged 7–9 years old.

### Population and sample

Our survey was conducted from August to December 2018, in the same randomly selected 50 villages chosen by the NTDCP for its impact survey in Kongwa District, Tanzania. The World Health Organization (WHO) recommends a sample size for trachoma surveys of sufficient numbers of children ages 1–9 years to detect a prevalence of TF of 4% with a confidence interval of +/- 2%, with a design effect of 2.71 in an evaluation unit (EU). An EU is considered a normal administrative unit for health care management, generally with a population between 100,000–250,000 persons [9,10]. Because the population of Kongwa District is about twice the EU size, the Tanzanian NTDCP doubled the sample size from about 970 children ages 1–9 years to about 1940, to be found in a random selection of 50 villages. We assumed that with 50 villages, we would need around 50 households per village, assuming a response rate of 75%, to achieve the suggested sample size of 1940. After randomly selecting a geographic neighborhood (*mtaa*) in a village, we performed a complete census, including age and sex, of all persons in the households in the *mtaa*. Using the census data, we randomly selected 50 households and enrolled all children ages 1–9 years from each household.

### Survey and sample collection

The primary outcome was clinical trachoma, as measured in the field and in photographs, as described below. The field grader was trained by a Global Trachoma Mapping Project trained grader (HM) and standardized by comparing grades on both photos and in the field. Using a flashlight and 2.5x magnifying binocular loupes, the trachoma field grader assessed the upper tarsal conjunctiva of both eyes in each child for the presence or absence of trachomatous inflammation—follicular (TF) and trachomatous inflammation—intense (TI) according to the WHO simplified grading scheme [11]. Photographs were also taken of the upper tarsal conjunctiva of at least one eye using a handheld Nikon D40 digital SLR camera in a manual setting, with a 105 mm f/2.8D Auto Focus Micro Nikkor lens (fully extended). The camera malfunctioned during the survey, so photographs were not available for all children.

The presence of infection with *C*. *trachomatis* was also determined. An ocular swab was collected from the left tarsal conjunctiva of each child using a sterile Dacron polyester-tipped swab. The swab was rolled across the conjunctiva twice to ensure that sufficient epithelial cells were obtained. The technician taking the swab wore gloves, did not touch the child or any other surface, and observed field protocol to avoid contamination. Every community had 2–4 “air” swabs, assigned at random to monitor for contamination, where swabs were waved in the air near a child. The air swabs were spiked with human DNA from buccal samples to avoid the air swabs being labeled as “invalid” during later processing. All swabs were stored in sterile tubes, kept in a cooler in the field, and refrigerated at 2–8°C for fewer than 30 days before being sent dry on cold packs to the laboratory at Muhimbili Medical Center (Dar es Salaam, Tanzania), where they were stored at -80°C until they were ready to be processed. The negative air controls were labeled and processed identically to the true samples, and laboratory staff were blinded to which swabs were samples versus controls.

The presence of antibodies to *C*. *trachomatis* antigen pgp3 was also assessed to determine the seropositivity status of the district. Finger prick blood was collected from each child onto filter paper with six circular extensions, each of which was calibrated to absorb 10 μL of whole blood. The blood spots were air dried, stored at -20°C, then shipped to the Centers for Disease Control and Prevention (Atlanta, Georgia, USA) for processing using the Luminex 100 system.

### Laboratory processing

Due to budget limitations, not all villages had swabs processed for infection. Only villages where TF was present had all the swabs processed. We tested pools (size: 4–5) of specimens using the Cepheid PCR platform (Cepheid, Sunnyvale, California) according to manufacturer’s directions. Cepheid tests for specimen adequacy by testing for human DNA in addition to *C*. *trachomatis* genetic material. The results of each pool test could be negative, positive, or invalid. If a pool tested negative, all the specimens in that pool were considered negative for *C*. *trachomatis*. If a pool tested positive, the pool was deconstructed, and the specimens were retested individually to identify the positive sample(s). If the result of a retest was positive, the sample was considered positive for *C*. *trachomatis*. Pools with invalid results were retested both as pools and as individual specimens to determine positive or negative final results [12]. All laboratory personnel were masked to the trachoma status and serologic status of the person providing the specimen.

The dried blood spot eluates were analyzed using a multiplex bead array and read on a Bio-Plex 200 system. A summary has been provided here, but additional details may be found elsewhere [13]. Chlamydial antigen pgp3 was expressed in a bacterial expression system and coupled to SeroMAP polystyrene beads (Luminex, Austin, Texas). Serum was eluted from the dried blood spots, and the resulting eluates were incubated with the pgp3-coupled beads. Biotinylated mouse anti-human total IgG (clone H2; Southern Biotech, Birmingham, Alabama) and IgG4 (clone HP6025; Invitrogen, South San Francisco, California) were added to each sample to detect the total anti-pgp3 IgG bound to the beads. Because the fluorescent protein conjugate R-phycoerythrin-labeled streptavidin (SAPE) (Invitrogen, South San Francisco, California) binds biotin [14], the subsequent measure of fluorescence served as a proxy measurement for total anti-pgp3 IgG bound to the beads in a given sample. SAPE was added to the wells, and fluorescence was measured using a Bio-Plex 200 instrument (Bio-Rad, Hercules, CA) running Bio-Plex Manager 6.0 software (Bio-Rad), which provided results as median fluorescence intensity minus background (MFI-BG)—in other words, the median fluorescence intensity of a sample minus the background intensity of a negative control well with just buffer. MFI-BG values were then interpreted as seropositive or seronegative based on a cutoff determined by a receiver operator characteristic (ROC) curve analysis using the J-index to maximize both sensitivity and specificity. For these data, the cutoff for seropositivity was MFI-BG = 1771. The laboratory personnel were masked to the trachoma status and infection status of the provider of the specimen.

### Photo grading

The photographs were sent to Johns Hopkins and graded for the presence or absence of TF and TI by two trachoma photo graders (MO and FN) using the WHO simplified grading scheme [11]. The training was supervised by a senior grader (SW) and consisted of a didactic course on the WHO simplified scheme, followed by open discussion of 100 images, including borderline cases and images difficult to grade. Each grader was gradually urged to take over calling the grade, followed by discussion. After the training, a test set of 60 images was graded independently by the trainees, and each achieved a kappa of 0.70 or better against the senior grader to be certified. At the end of their training, the two graders had strong agreement between themselves, with a kappa of 0.92 (*95% Confidence Interval*: 0.81–1.00). The graders were masked to all participant identifying information, including field grades and grades from the other photo grader. Disagreements were openly adjudicated with the senior grader, and the final adjudicated grade for the image was used.

### Data analyses

If a child had at least one eye that had TF, the child was considered to have TF. Infection was defined as a conjunctival swab specimen that was positive by Cepheid testing. Seropositivity was defined as an MFI-BG greater than 1771. Prevalence of TF was calculated as the proportion of children positive for TF in all children surveyed, and the confidence interval was adjusted for clustering by village using generalized estimating equations. Estimated TF prevalence was standardized to the population-level age distribution of children ages 1–9 years in Kongwa District using the weights from the 2012 census.

Contingency tables were used to analyze frequencies of TF, infection, and seropositivity. Logistic and linear regression models as appropriate were used to test the significance of the associations between the main outcomes and demographic characteristics. The generalized estimated equation (GEE) approach was used to account for the within-village correlation. MFI-BG values that were negative or 0 were converted to 1 for the log(MFI-BG) calculations. Intraclass correlation coefficients (ICC) were estimated using the variance of the random intercept from a logistic model, with TF and seropositivity as the outcomes. The relationship between village-level TF prevalence and seropositivity prevalence was assessed with a Pearson correlation coefficient. Confidence intervals and p-values for overall TF prevalence and seropositivity were adjusted for clustering. The data were deidentified, and analyses were conducted using SAS 9.04.01M6 software.

Kongwa District is subdivided by the government into 22 local wards. We mapped the location of each village in our sample, the village-level TF prevalences, and the population of the wards in Kongwa District. The ward shapefile for Kongwa District was generated from the 2012 Tanzanian Census [15]. Each village was mapped with GPS coordinates of the village center, and the GPS coordinates and ward populations came from a 2017 survey of the entire district. The map was generated with ArcGIS Esri 10.7.1 software.

Our raw data are available in S1 Data, and the STROBE checklist may be found in S2 Data.

## Results

A total of 3048 children were enumerated, of whom 2401 children ages 1–9 years were surveyed. Of the remaining 647 children, 581 (90%) were traveling on the days of the survey in their villages, 59 (9%) refused, and the remaining 7 (1%) had died or could not be found. A total of 2399 of the children surveyed had both eyes graded in the field. The remaining 2 children each received a grade for one eye, with the other eye not available. All children provided dried blood spots for serological analysis, but 10 (0.4%) specimens were lost and not processed.

The overall TF prevalence was 7.1% (*95% CI*: 5.6%-8.9%). There was no statistically significant difference in TF by sex, but TF did differ by age, with the lowest rates in children ages 7–9 years (Table 1). After age-adjusting the TF prevalence for the population-level age distribution in Kongwa District, the estimate of overall prevalence rose slightly to 7.3%.

**Table 1:** Prevalence of field grade of trachoma by the characteristics of the sample of children ages 1–9 years in Kongwa District, Tanzania.

Characteristic		N (% of Total)	N TF(+)	% Prevalence	P-value
**Age**	1–3	773 (32.2%)	81	10.5%	<0.0001
	4–6	837 (34.9%)	62	7.4%	
	7–9	791 (32.9%)	27	3.4%	
**Sex**	Female	1219 (50.8%)	87	7.1%	0.9146
	Male	1182 (49.2%)	83	7.0%	
**Total**		2401	170	7.1%	-

### TF by field versus photo grading

We corroborated the field grading with the photo grading. Photographs were taken in both eyes of 707 children, and 701 had gradable photographs. The percent agreement between field and photo grading was 95%, and the kappa between field and photo grading was 0.69 (*95% CI*: 0.60–0.78) (Table 2). The agreement was good although the field grader had a slight tendency to call more TF in the field (10.70%) than the photo graders did for the images (8.70%) in this subset of children.

**Table 2:** Relationship between field and photo grading of trachoma in children ages 1–9 years (N = 701)^a^.

Field Grade	Photo Grade		Total
	*TF(-)*	*TF(+)*	
***TF(-)***	614 (87.6%)	12 (1.7%)	626
***TF(+)***	26 (3.7%)	49 (7.0%)	75
**Total**	640	61	701
**Kappa** = 0.69 (*95% CI*: 0.60–0.78)			

^a^ Photos of both eyes were taken in 707 children, but 4 children had ungradable photos for both eyes and 2 children had corrupted data files for the images of the right eye. These grades are reported at the child level, so if a child had at least one eye with TF, the child was considered to have TF.

### TF and infection

1720 children from 36 villages had a test for infection, with 16 children yielding invalid results because the samples did not contain sufficient human DNA. One of the ocular swab air controls tested positive, suggesting that some of the specimens collected on that day or run on that day in the lab may have been contaminated. The children before and after the child with the air swab tested negative for infection, and the child associated with the air swab had neither TF nor TI, so we suspected contamination in the lab. All specimens from that day in the lab were re-run, and all but one were negative, confirming lab contamination. The lab was thoroughly disinfected prior to re-running any further specimens, the protocol was reviewed, and no further air swabs were positive moving forward. Of the 1704 children with infection data, 21.5% of those with TF were positive for infection, and 1.6% of children without TF were positive for infection (p = 0.0010) (Table 3).

**Table 3:** Relationship of TF in children ages 1–9 years with a positive test of infection (N = 1704).

TF Status	N	N Infection(+)	% Infection(+)	P-value
TF(-)	1541	25	1.6%	0.0010
TF(+)	163	35	21.5%	
**Total**	1704	60	3.5%	-

### Seropositivity

**Fig 1:**
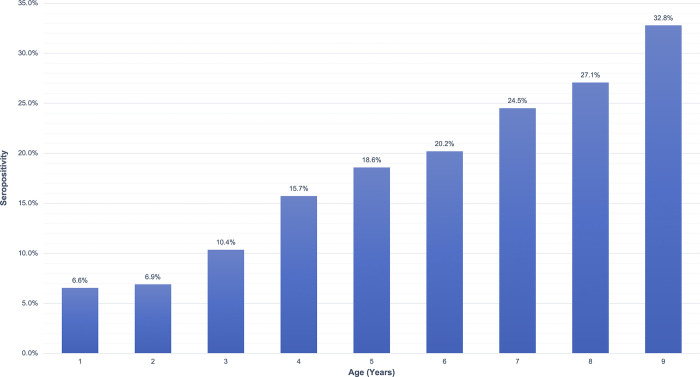
Seropositivity by age in children ages 1–9 years in Kongwa District, Tanzania.

**Fig 2:**
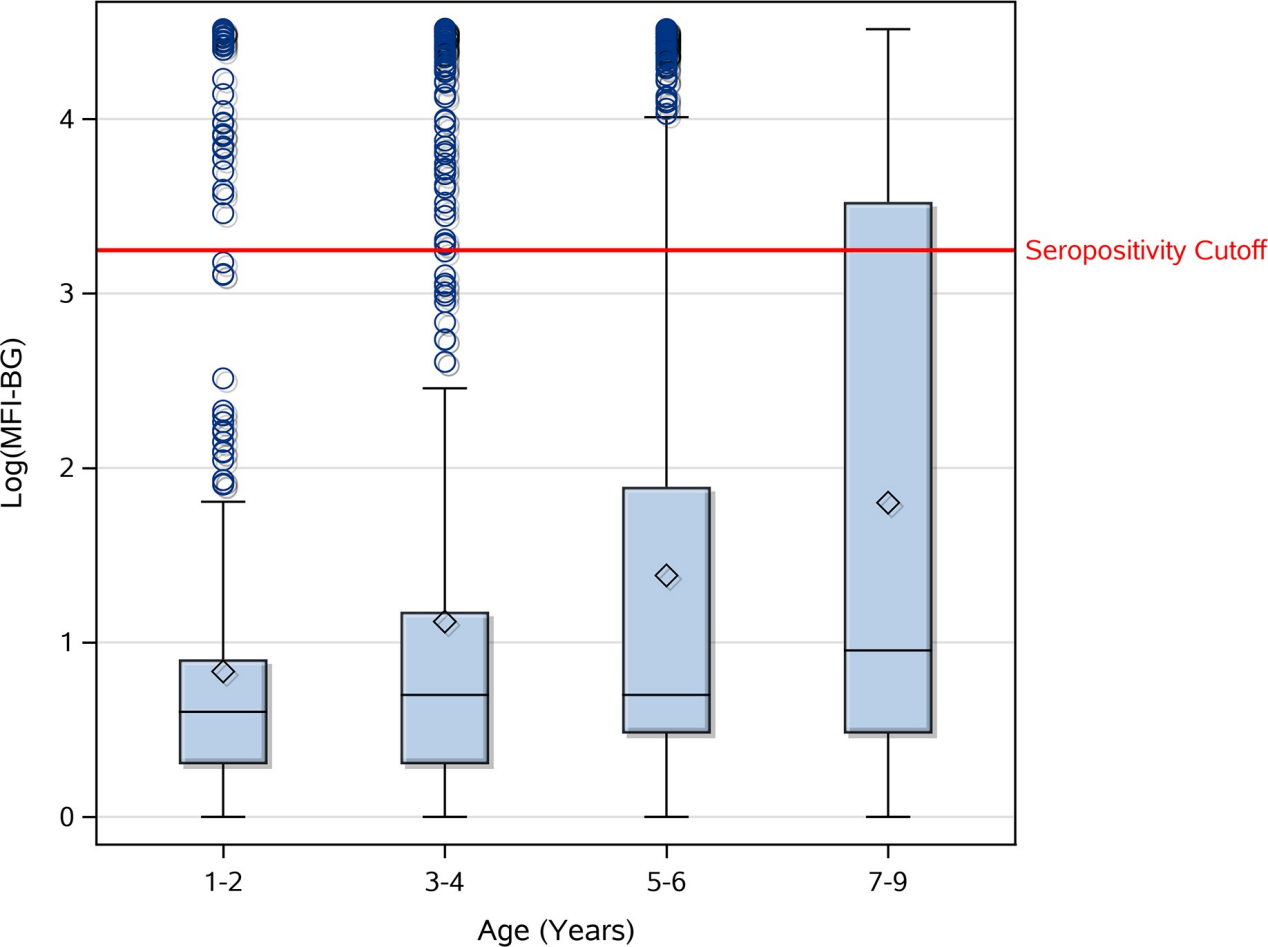
Anti-Pgp3 antibody levels [log(MFI-BG)] by age. The horizontal line within each box represents the median, and the diamond represents the mean.

The overall seropositivity among the 2391 children with data was 18.2% (*95% CI*: 14.8%-22.1%). There was no significant difference in seropositivity by sex (p = 0.5680). However, seroprevalence increased significantly with age (p = <0.0001) (Fig 1). The children ages 1–2 years, who were born after the cessation of MDA, had an average seroprevalence of 6.7%, and children age 3 years had an average seroprevalence of 10.4%, which increased 1–6% with each additional year of life. Mean log(MFI-BG) increased with age (p = <0.0001) (Fig 2), as well as with presence of TF (p = 0.0003) and infection (p = <0.0001).

Of note, all but 5 of the children with infection were also seropositive. Of the 5 with infection who were seronegative, none had TF either.

### Village-level TF prevalence groups and seropositivity

**Fig 3:**
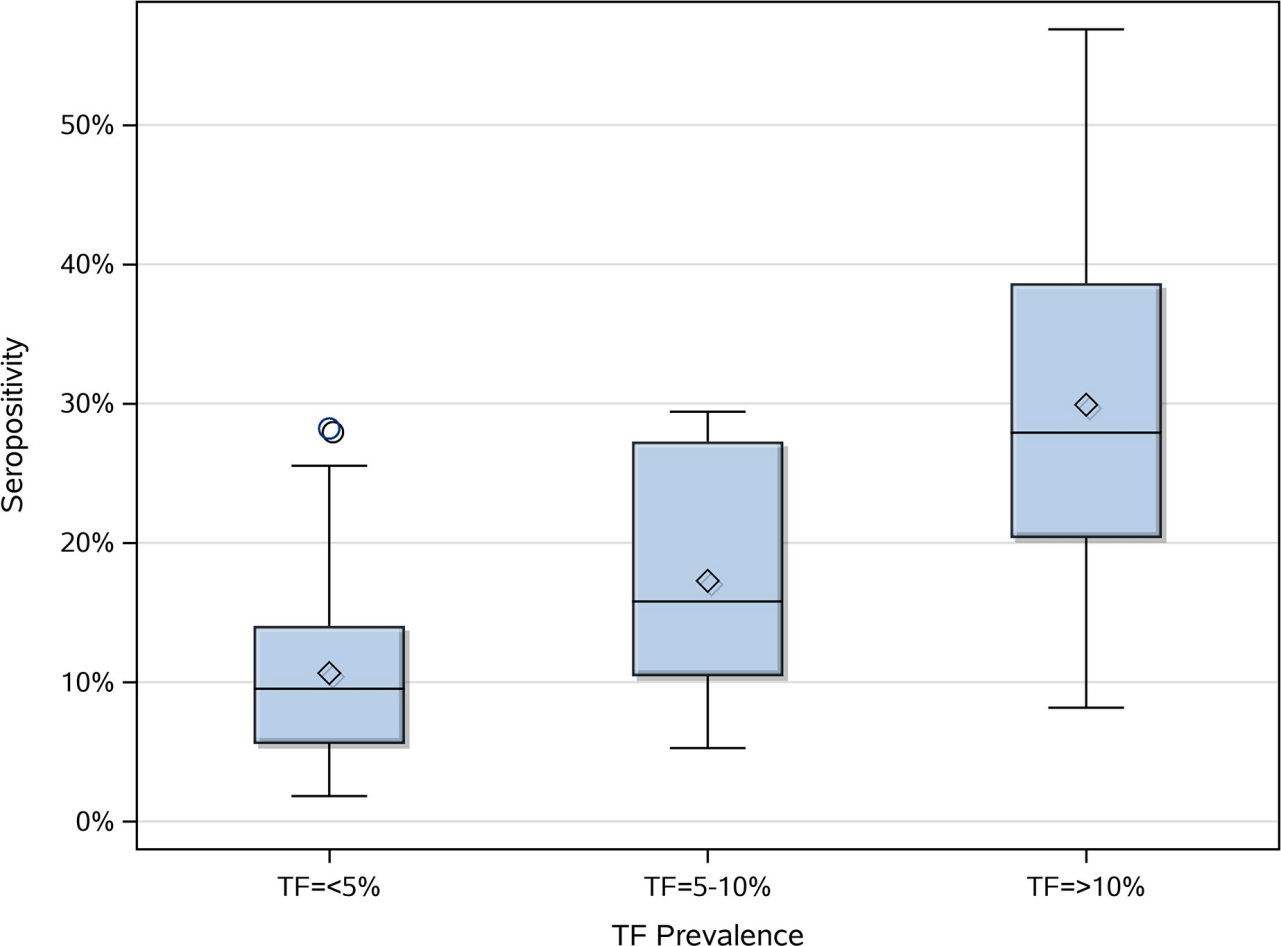
Village-level percent seropositivity by category of village TF endemicity in children ages 1–9 years. The horizontal line within each box represents the median, and the diamond represents the mean.

19 of the 50 villages surveyed had TF prevalence <5%, 15 of the villages had TF prevalence 5–10%, and 16 of the villages had TF prevalence >10%. Seropositivity increased significantly with the degree of TF endemicity (p = <0.0001) (Fig 3). Age distributions between these villages of different TF endemicity levels were not significantly different (p = 0.4845), supporting the conclusion that the differences in seropositivity by village degree of TF endemicity were not due to differing age distributions between villages.

The ICC calculated for seropositivity was 0.18 (*95% CI*: 0.10–0.26) (p = <0.0001) and the ICC for TF was 0.15 (*95% CI*: 0.05–0.24) (p = 0.0036), suggesting clustering of TF and seropositivity within villages. The Pearson correlation coefficient between TF prevalence and seropositivity at the village level was 0.74 (p = <0.0001), indicating a strong positive correlation between village-level TF prevalence and seropositivity.

Investigation of the seropositivity in children ages 1–2 years by the category of village-level TF prevalence in children ages 1–9 years is shown in Table 4. Among the 8 villages with no TF, only 1 village had any children ages 1–2 years with positive serology. In the 16 villages with TF >10% in 1–9 year olds, the mean prevalence of seropositivity in 1–2 year olds was 12.3%.

**Table 4:** Prevalence of seropositivity in children ages 1–2 years by village-level TF prevalence in children ages 1–9 years.

Village-level TF Prevalence in Children Ages 1–9 Years	Number of Villages	Proportion of Villages with All Children Ages 1–2 Years Seronegative	% Prevalence of Seropositivity in Children Ages 1–2 Years	
			*Mean (SD)*	*Median (IQR)*
0%	8	7/8 (87.5%)	0.9% (2.5)	0.0% (0.0, 0.0)
>0% to <5%	11	8/11 (72.7%)	4.3% (7.7)	0.0% (0.0, 11.1)
5% to 10%	15	9/15 (60.0%)	5.5% (7.6)	0.0% (0.0, 12.5)
>10%	16	9/16 (56.3%)	12.3% (16.3)	0.0% (0.0, 27.3)

**Fig 4:**
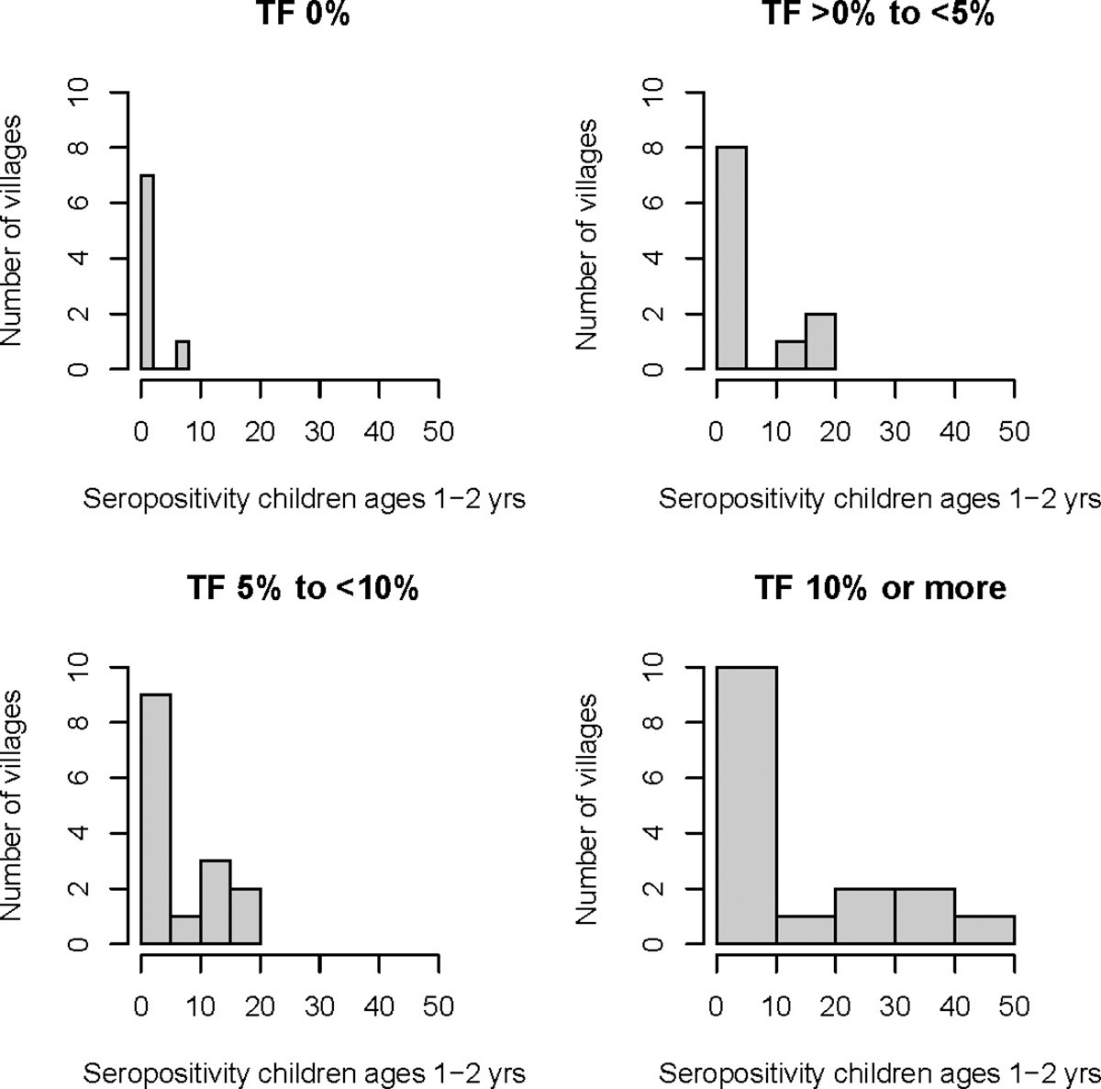
Number of villages with various percent seropositivities in children ages 1–2 years, categorized by overall village-level TF prevalence in children ages 1–9 years.

In the 17 villages where seropositivity in children ages 1–2 years was >0%, all but 3 of the villages had a seroprevalence >10% (Fig 4), suggesting the locations where most of the transmission was likely occurring.

### Geographic distribution of TF by village

**Fig 5:**
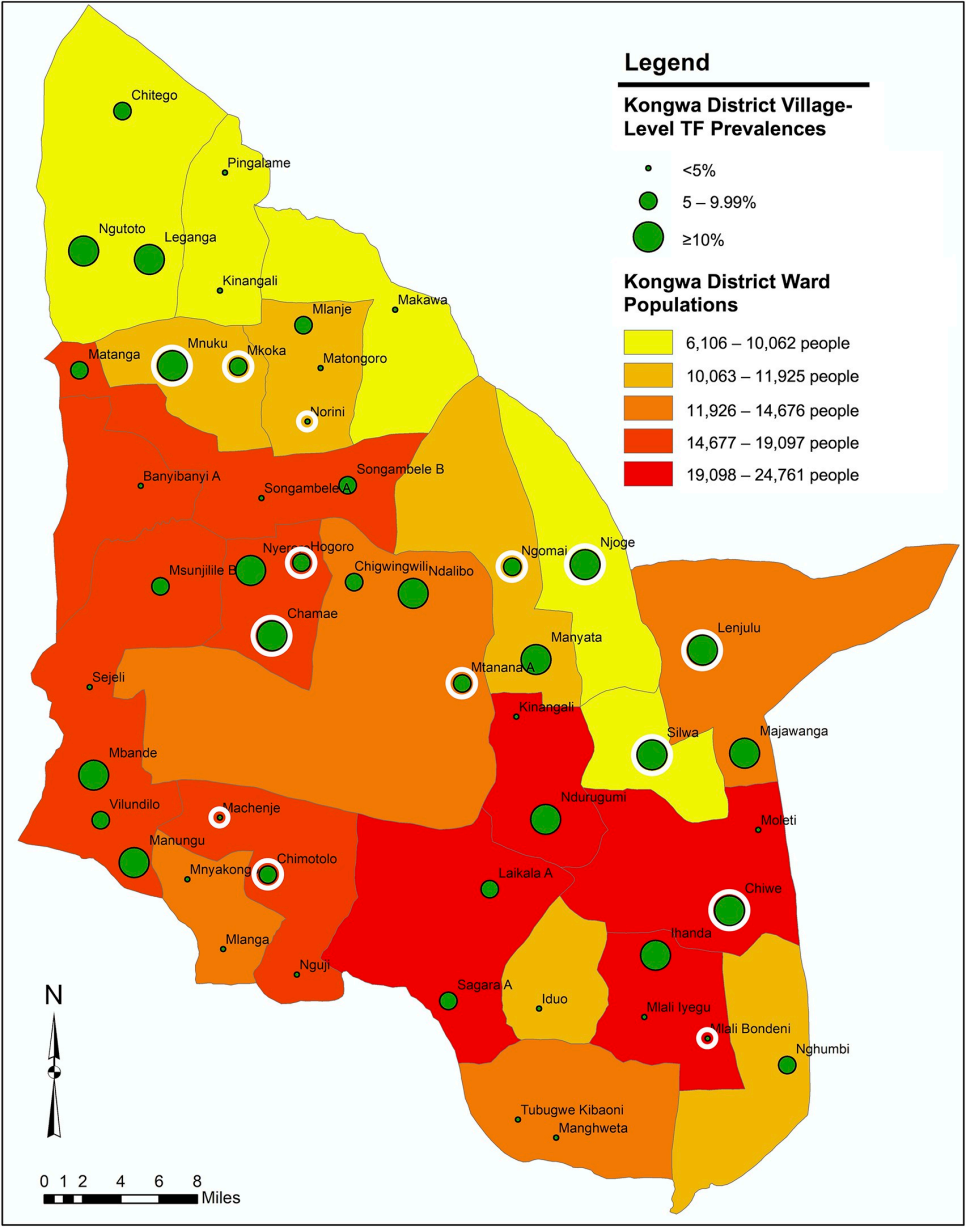
Geographic distribution of village-level TF prevalence in children ages 1–9 years in Kongwa District, Tanzania. The circular white borders indicate villages that also had percent seropositivity >10% in children ages 1–2 years. Base map from Humanitarian Data Exchange: https://data.humdata.org/dataset/2012-census-tanzania-wards-shapefiles.

The 31 villages with TF prevalence >5% in children ages 1–9 years were dispersed throughout the district, and there was no clear region of the district where most of the villages with high TF prevalence were located. Similarly, the 14 villages with percent seropositivity >10% in children ages 1–2 years were scattered throughout the district (Fig 5).

## Discussion

The overall TF prevalence in Kongwa District 2 years post-MDA was 7.1% (*95% CI*: 5.6%-8.9%) and the age-adjusted TF prevalence was 7.3%, both of which were greater than the established threshold of 5% and suggested that trachoma had re-emerged. We used photographic images, as well as a test of infection, to corroborate the field grading of TF. We demonstrated a good correlation between photo and field grading, with a kappa of 0.69. Of the 701 children with valid photo data for both eyes, field and photo graders assigned the same TF grade 95% of the time, only differing over 38 children. Even if the field grader had overcalled TF, we estimated that the prevalence would change only by a maximum of 2% and would thus still result in a TF rate greater than 5%. Infection rate was also higher in children with TF compared to children without TF. The percent of TF-positive children with infection (21.5%) was slightly less than that reported in another study of Kongwa District [16] but still indicated the presence of infection in cases of TF. These two tools were used to support the field grade, and based on these data, we reject our hypothesis that TF prevalence would be less than 5% in children ages 1–9 years in the district 2 years after the last round of MDA.

Especially noteworthy is that the TF prevalence was highest in the 1–3 year olds (10.5%), who were largely born after the last round of MDA. The youngest age group is of special interest as we turn to the serologic data. As expected, seropositivity increased with age, reflecting previous exposure to *C*. *trachomatis* when the district rates were higher. We found seropositivity of 18.3% in children ages 4–6 years, and 28.1% in children ages 7–9 years. However, if transmission had been interrupted following the cessation of MDA after the impact survey found TF <5%, we would have seen little to no seropositivity in the 1–2 year olds, instead of the 6.7% we observed. Moreover, the log(MFI-BG) for the 1–2 year olds revealed a clear pattern of seropositivity, with only 3 cases near the cutoff and the rest clearly seronegative or seropositive.

It is unlikely that the seropositivity in these children was due to maternal antibodies to urogenital chlamydia or antibodies generated in response to neonatal chlamydial infection. Although current data do not exist for Kongwa District, genital chlamydia in Kongwa seems to be rare. Thus, we do not feel that the rates in the 1 year olds reflect lingering maternal antibodies, which in any case, rarely last beyond 6 months. While it appears that some children develop antibodies in response to an ocular conjunctivitis or respiratory infection after exposure during birth to a maternal genital infection [17–19], the low suspicion of genital chlamydia in Kongwa makes this seem less likely to be occurring here.

It is difficult to compare the actual seroprevalence data to other studies, where the methods for antibody detection and defining seropositivity, as well as the bead sets used for multiplex bead array, were different. In our previous data from Kongwa District, the overall seropositivity was much higher (31%) than observed here even in the oldest children [8]. If we examine the pattern of seropositivity by age, we can observe that the seropositivity rate in the 1–2 year olds was higher than the rates in studies that used a similar technique and method for calculating seropositivity but were conducted in districts that had achieved the elimination criterion of TF <5% in children ages 1–9 years. For instance, a study in Nepal found overall 2.4% seropositivity and no age-specific increase in seropositivity [20], and a study in Kilosa District, Tanzania, found only a very small age-specific increase with an overall rate of 7.5% [6]. A recent study in Ethiopia used a very similar serological protocol to ours, making it easier to compare our data to theirs. In Alefa District approximately 2.5 years post-MDA, where TF prevalence was found to be 3.2%, the 1–2 year olds (born after cessation of MDA) had an anti-pgp3 seropositivity of about 0–1%. Given that Alefa had achieved the elimination criterion of TF <5% in children ages 1–9 years, we would have expected the seropositivity for our 1–2 year old age group born post-MDA to be similar to that value of 0–1%, rather than 6.7%. In two districts with high TF prevalences of 14.7% (Dera) and 37% (Andabet), although they had last received MDA only about 8 months prior, the 1 year olds had seropositivities around 3% (Dera) and 8% (Andabet). Thus, it was unexpected that the seropositivity from our 1–2 year olds more closely resembled the seropositivities of the youngest children in these trachoma-endemic districts [21].

In this study, we might have expected to observe the age-specific increase in seropositivity after age 3 years because it had only been 2 years since cessation of MDA. Absence of trachoma and infection has been associated with seroreversion, which would lead to a collapse in the age-specific seroprevalence over time [8]. However, in the presence of transmission, seroreversion is far less likely, and an increase due to ongoing seroconversion is more likely. The similarity between the two seropositivities in the 1 and 2 year olds suggests that transmission had been low in the year immediately following cessation of MDA, but began to increase again 1 year post-MDA. If, as is likely, serology reflects cumulative exposure to infection and thus trachoma, then the fact that both ages 1 and 2 year olds had the same percent seropositivity suggests that children of both ages were exposed for the same amount of time, indicating that TF likely began to re-emerge after the birth of the 1 year olds, and thus 1 year post-MDA. This supposition is supported by other data on infection spreading following MDA, which suggests it takes longer than 6 months to spread outside the household to neighboring households [22]. The data also support the guideline of waiting to undertake an impact survey until 1 year after MDA.

The 5 children who had infection with *C*. *trachomatis* but were seronegative were also negative for TF. It is likely they had new infections and had not yet developed a detectable circulating antibody response or evidence of disease. It is also possible that some had false positive infection results, although specificity for the Cepheid platform has been reported as 100% [12].

Of note, although the overall prevalence of TF in Kongwa District was 7.1%, there was evidence of heterogeneity among the villages. Of the 50 villages surveyed, 16 had estimated TF prevalence >10%, while 19 villages had an estimated TF prevalence <5%. Although we did not perform a formal clustering analysis, the villages with TF prevalence >5% seemed to be scattered throughout the district, suggesting a more complex pattern of transmission than a single entry point. While we recognize that the survey was powered to estimate prevalence at the EU level, we felt it was important to also use the data at a finer level—in this case, by grouping village-level data into categories—to understand if there was evidence of re-emergence from a particular geographic area and to better understand the relationship of seropositivity to clinical trachoma.

There were some limitations to this study. Because of budgetary constraints, we prioritized testing ocular swab specimens from villages with trachoma to be sure we sampled enough cases of trachoma to correlate infection with TF. The data were informative for demonstrating that clinical determinations of TF were highly associated with infection, but we had insufficient data to determine a prevalence of infection associated with re-emergence. Ideally, we would have also had serologic data from the initial post-MDA impact survey, using the same procedures and bead set, to determine if there had been a change over the ensuing 2 years, as that data on seroreversion and seroconversion would have made a stronger case for re-emergence. In addition, for this study, the seropositivity threshold was much higher than it has been historically, which resulted in a lower seroprevalence than expected. This meant that we could not reliably compare seropositivity longitudinally to previous reports in Kongwa District, which is one of the potential benefits of antibody testing for surveillance. Nevertheless, we were still able to study the age curve and derive some conclusions using the youngest age group, which further corroborated the likelihood of re-emergence. More work on standardization of antibody testing, especially a set of standard operating procedures for laboratory analysis of dried blood spots to detect antibodies against *C*. *trachomatis*, should be developed and circulated. A positive control chimeric antibody to set seropositivity cut-offs is also essential. Additionally, because we only had village-level TF prevalence data for a sample of villages, rather than for all 92 villages in Kongwa District, it would have been difficult to determine clustering of potential hot spots for re-emergence. Nevertheless, based on our map, we were able to demonstrate that the 31 villages with TF >5% were distributed all throughout the district, suggesting the need for district-wide resumption of MDA and F and E activities.

The strengths of the study included the large sample size, which made the estimates of TF prevalence and seropositivity very robust. It also permitted us to undertake some exploratory analyses on the heterogeneity of the re-emergence. Another strength was the use of air controls during swab acquisition, which enabled us to determine the instance of contamination in the laboratory. Proper containment and re-analysis measures were instituted which avoided data loss and/or false positive specimens. This was a critical quality control metric, and although it adds to expense, it should be part of every protocol where specimens are collected. Finally, careful standardization of photo graders, as measured by grader agreement, reduced the likelihood of inter-grader variability and added to the reliability of the determination of TF from photographs, which was a critical check on the field grading.

In conclusion, our study found value in using a test of infection and photography as adjunct tools to corroborate evidence of re-emergent trachoma in Kongwa District. The serologic data not only provided support for this conclusion, but by examination of the youngest age group, also helped to pinpoint the likely timing of re-emergence. We found that 2 years post-MDA was a reasonable interval to detect re-emergence, and these data suggest that the timing of the NTDCP’s impact survey 6 months post-MDA was likely too early to have picked up this re-emergence. Our data also supported the validity of serology as a potential surveillance tool for trachoma re-emergence. Serological testing is promising in large part because of its convenience and the ability to monitor for re-emergence of trachoma while simultaneously monitoring for other neglected tropical diseases via multiplex bead array. Our data suggest that not only would serological testing be cost-efficient, it could also be effective for early detection of trachoma re-emergence if used in age groups where transmission is not expected.

## Supporting information

S1 Data Excel spreadsheet with raw data. (XLSX)

S2 Data STROBE checklist for cross-sectional studies. (DOC)
