# Supplementary material for: Serology, infection, and clinical trachoma as tools in prevalence surveys for re-emergence of trachoma in a formerly hyperendemic district
Source: PLoS Negl Trop Dis. 2021 Apr 16;15(4):e0009343. doi: 10.1371/journal.pntd.0009343 (PMC8081338; doi:10.1371/journal.pntd.0009343)
Supplement: S2 Data — (DOC) [file pntd.0009343.s002.doc]

STROBE Statement—Checklist of items that should be included in reports of ***cross-sectional studies***

|  | Item No | Recommendation |
| --- | --- | --- |
| **Title and abstract** | 1 | (*a*) Indicate the study’s design with a commonly used term in the title or the abstract  Page 1 Title (prevalence surveys) |
|  |  | (*b*) Provide in the abstract an informative and balanced summary of what was done and what was found  Page 3 lines 50-54 |
| Introduction | | |
| Background/rationale | 2 | Explain the scientific background and rationale for the investigation being reported  Page 4-5 line 88-101 |
| Objectives | 3 | State specific objectives, including any prespecified hypotheses  Page 5-6 line 111-114 |
| Methods | | |
| Study design | 4 | Present key elements of study design early in the paper  Page 5 line 103-111 |
| Setting | 5 | Describe the setting, locations, and relevant dates, including periods of recruitment, exposure, follow-up, and data collection  Page 6 127-135 |
| Participants | 6 | (*a*) Give the eligibility criteria, and the sources and methods of selection of participants  Page 6-7 line 127-140 |
| Variables | 7 | Clearly define all outcomes, exposures, predictors, potential confounders, and effect modifiers. Give diagnostic criteria, if applicable  Page 6-9 line 144-224 |
| Data sources/ measurement | 8* | For each variable of interest, give sources of data and details of methods of assessment (measurement). Describe comparability of assessment methods if there is more than one group  Page 7-10 line 144-224 |
| Bias | 9 | Describe any efforts to address potential sources of bias  Page 7-8 line 159-167; Page 9 line 186-188; page 10 line 207-209 and line 221-224. |
| Study size | 10 | Explain how the study size was arrived at  Page 6-7 line 127-140 |
| Quantitative variables | 11 | Explain how quantitative variables were handled in the analyses. If applicable, describe which groupings were chosen and why  Page 10-11 lines 228-246 |
| Statistical methods | 12 | (*a*) Describe all statistical methods, including those used to control for confounding  Page 10-11 lines 228-246 |
|  |  | (*b*) Describe any methods used to examine subgroups and interactions  Page 11 lines 236-239, 242-244; Page 11-12 248-253 |
|  |  | (*c*) Explain how missing data were addressed: very low missing data so noted  Page 12 line 260-265; page 13 line 286-287 (Table 2) |
|  |  | (*d*) If applicable, describe analytical methods taking account of sampling strategy  Page 11 line 232-234 |
|  |  | (*e*) Describe any sensitivity analyses |
| Results | | |
| Participants | 13* | (a) Report numbers of individuals at each stage of study—eg numbers potentially eligible, examined for eligibility, confirmed eligible, included in the study, completing follow-up, and analysed  Page 12 line 262-264 |
|  |  | (b) Give reasons for non-participation at each stage  Page 12 line 261-264 |
|  |  | (c) Consider use of a flow diagram |
| Descriptive data | 14* | (a) Give characteristics of study participants (eg demographic, clinical, social) and information on exposures and potential confounders  Page 13 Table 1 |
|  |  | (b) Indicate number of participants with missing data for each variable of interest  Page 12 line 264-265; page 13 line 286-287 (Table 2); page 14 line 293-294 |
| Outcome data | 15* | Report numbers of outcome events or summary measures  Page 12 line 267; page 12 line 278-280; Page 14 line 310-311 |
| Main results | 16 | (*a*) Give unadjusted estimates and, if applicable, confounder-adjusted estimates and their precision (eg, 95% confidence interval). Make clear which confounders were adjusted for and why they were included  Page 12 line 267-270; page 13 line 278-280; page 15 line 310-311 |
|  |  | (*b*) Report category boundaries when continuous variables were categorized  Page 11 line 239-240 |
|  |  | (*c*) If relevant, consider translating estimates of relative risk into absolute risk for a meaningful time period |
| Other analyses | 17 | Report other analyses done—eg analyses of subgroups and interactions, and sensitivity analyses  Tables 2-4, Figures 1-5 |
| Discussion | | |
| Key results | 18 | Summarise key results with reference to study objectives  Page 17-18 line 373-375, line 388-392 |
| Limitations | 19 | Discuss limitations of the study, taking into account sources of potential bias or imprecision. Discuss both direction and magnitude of any potential bias  Page 21-22 line 459-480 |
| Interpretation | 20 | Give a cautious overall interpretation of results considering objectives, limitations, multiplicity of analyses, results from similar studies, and other relevant evidence  Page 23 line 493-504 |
| Generalisability | 21 | Discuss the generalisability (external validity) of the study results  Page 23 line 500-504 |
| Other information | | |
| Funding | 22 | Give the source of funding and the role of the funders for the present study and, if applicable, for the original study on which the present article is based |
